# Low-Cost Dual-Frequency GNSS Receivers and Antennas for Surveying in Urban Areas

**DOI:** 10.3390/s23052861

**Published:** 2023-03-06

**Authors:** Veton Hamza, Bojan Stopar, Oskar Sterle, Polona Pavlovčič-Prešeren

**Affiliations:** Faculty of Civil and Geodetic Engineering, University of Ljubljana, 1000 Ljubljana, Slovenia

**Keywords:** GNSS, surveying, low-cost receivers, low-cost antennas

## Abstract

Low-cost dual-frequency global navigation satellite system (GNSS) receivers have recently been tested in various positioning applications. Considering that these sensors can now provide high positioning accuracy at a lower cost, they can be considered an alternative to high-quality geodetic GNSS devices. The main objectives of this work were to analyze the differences between geodetic and low-cost calibrated antennas on the quality of observations from low-cost GNSS receivers and to evaluate the performance of low-cost GNSS devices in urban areas. In this study, a simple RTK2B V1 board u-blox ZED-F9P (Thalwil, Switzerland) was tested in combination with a low-cost calibrated and geodetic antenna in open-sky and adverse conditions in urban areas, while a high-quality geodetic GNSS device was used as a reference for comparison. The results of the observation quality check show that low-cost GNSS instruments have a lower carrier-to-noise ratio (C/N_0_) than geodetic instruments, especially in the urban areas where the difference is larger and in favor of the geodetic GNSS instruments. The root-mean-square error (RMSE) of the multipath error in the open sky is twice as high for low-cost as for geodetic instruments, while this difference is up to four times greater in urban areas. The use of a geodetic GNSS antenna does not show a significant improvement in the C/N_0_ and multipath of low-cost GNSS receivers. However, the ambiguity fix ratio is larger when geodetic antennas are used, with a difference of 1.5% and 18.4% for the open-sky and urban conditions, respectively. It should be noted that float solutions may become more evident when low-cost equipment is used, especially for short sessions and in urban areas with more multipath. In relative positioning mode, low-cost GNSS devices were able to provide horizontal accuracy lower than 10 mm in urban areas in 85% of sessions, while the vertical and spatial accuracy was lower than 15 mm in 82.5% and 77.5% of the sessions, respectively. In the open sky, low-cost GNSS receivers achieve a horizontal, vertical, and spatial accuracy of 5 mm for all sessions considered. In RTK mode, positioning accuracy varies between 10–30 mm in the open-sky and urban areas, while better performance is demonstrated for the former.

## 1. Introduction

Global navigation satellite system (GNSS) technology has been used for many years for various navigation (air, water, and road transport), scientific (earth observation, weather forecasting, establishment of national coordinate systems, timing), monitoring (engineering structures, natural hazards), surveying, and many others purposes [1,2,3,4,5,6,7]. Geodetic GNSS devices are widely used in surveying applications, where positioning accuracy requirements can vary from millimeters to decimeters.

For many users and applications, high-quality geodetic GNSS instruments may turn out to be expensive. Instead, low-cost dual-frequency GNSS receivers, which have been available for the last few years only, are seen as an alternative solution. With the development of GNSS, more signals and constellations become available and can be tracked by low-cost GNSS receivers [8]. The advent of calibrated and choke ring low-cost antennas makes those GNSS devices more competitive with geodetic ones that are much more expensive [9].

Low-cost dual-frequency GNSS receivers were tested by researchers in relative, absolute, and real-time kinematic (RTK) positioning modes in both static and kinematic tests, and satisfactory positioning performance was obtained [10,11,12,13,14,15,16,17,18,19,20,21,22,23,24]. The performance of these devices in combination with patch antennas was evaluated in urban areas, considering RTK [19], static, relative, and absolute positioning modes [25,26].

Krietmeyer et al. [11] used low-cost GNSS receivers and different geodetic and low-cost antennas to estimate the zenith tropospheric delay (ZTD). The authors concluded that ZTD from low-cost GNSS devices is comparable to ZTD determined on a basis of high-ended geodetic devices. In the case of [15], the zero baseline and short baseline tests were performed to evaluate the quality of the u-blox receiver and the calibrated and uncalibrated low-cost antennas. The zero baseline test shows that ZED-F9P has low noise, in the millimeter range, while the results from the short baseline test prove that the calibrated antennas have better performance in comparison to the low-cost uncalibrated antennas used. 

In the study in [14], low-cost GNSS receivers were tested for geodetic monitoring purposes at short baselines, while low-cost and geodetic GNSS receivers were used as reference stations. Results indicate that 10 mm spatial displacements can be detected in half-hour sessions, even when only low-cost GNSS devices are used. In precise point positioning (PPP) mode, the dual-frequency GNSS receivers were able to detect movements in the magnitude of 20 mm [12]. Those devices were also used for monitoring engineering buildings over short baselines in RTK and static relative mode, and excellent positioning performance was achieved [27]. In the case of [28], such cost-effective equipment was capable of monitoring slow deformations of natural objects with high accuracy. Other researchers found that low-cost GNSS devices are able to provide high position accuracy in static PPP mode when the sampling rate is 1s or less. Accuracy was compromised when using data recorded at 5, 15, and 30 s [13]. 

The u-blox dual-frequency GNSS chip ZED-F9P and the ANN-MB-00 patch antenna have been tested in open-sky and urban conditions for surveying purposes. In open-sky conditions and for short baselines, accuracy in the centimeter range was achieved in relative positioning mode. However, in the case of urban areas, accuracy was 20 and 15 mm for the horizontal and vertical components, respectively. In RTK mode, the accuracy specified by the manufacturer was not achieved. Uncertainties were larger for both components due to phase center offset (PCO) and phase center variations (PCV) in the patch antennas used [25]. Rosendo-Andrade [26] used the same equipment for surveying in urban areas in relative and absolute positioning modes. The results show that both methods can be used for surveying in urban areas in case of optimal surveying conditions. The static relative method had higher accuracy and was suitable for surveying where the accuracy requirements are in the centimeter range. The PPP method was recommended only in the case where the open sky is guaranteed with fewer obstacles for satellite signals. In the study [19], the RTK engine of the u-blox C099-F9P (Thalwil, Switzerland)application board was tested in the open sky, the near-open-sky, in forests, and in urban conditions. The authors conclude that the low-cost receiver works well in the open sky, while the use of a geodetic antenna can significantly improve its positioning performance.

The number of scientific articles dealing with the evaluation of low-cost dual-frequency GNSS receivers for surveying purposes in urban areas is very limited. In most cases, open-sky conditions and patch antennas have been used whose PCO/PCV are unknown and are reported to mostly impact height components [14,25]. 

The main objectives of this work are two-fold. The first is to analyze the differences between geodetic and low-cost calibrated antennas on the quality of observations from low-cost GNSS receivers and the second is to evaluate the performance of low-cost GNSS devices in urban areas. The structure of the paper has been designed as follows: an overview of various tests of low-cost GNSS equipment is presented, with a particular focus on examining the performance of this equipment in urban areas (Section 1). The study area, equipment used, and methods are shown in Section 2. Then, the results are presented and discussed (Section 3). Finally, the conclusions from the study are listed (Section 4).

## 2. Materials and Methods

### 2.1. GNSS Devices 

**Figure 1:**
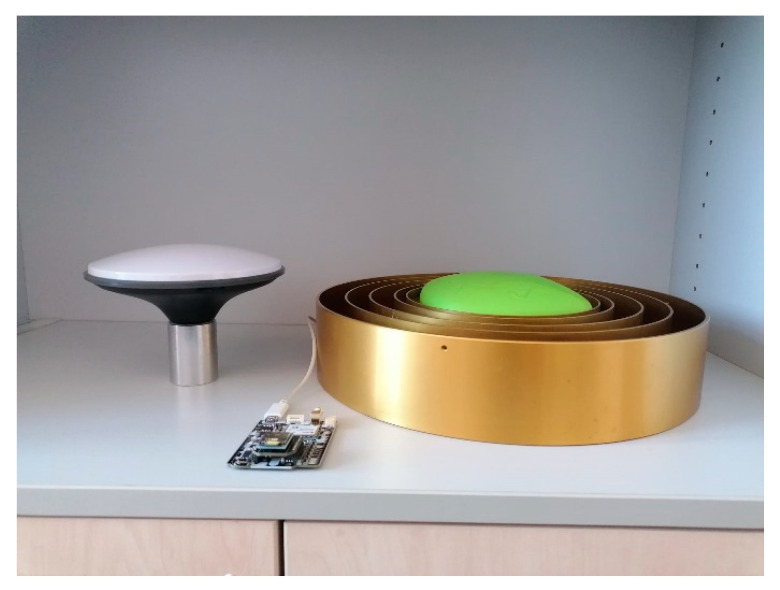
Low-cost GNSS receiver simpleRTK2B V1 (in front), Survey calibrated low-cost antenna (left), and Javad RingAnt-G3T geodetic antenna (right).

In this work, the low-cost simple RTK2B V1 GNSS board (~200 EUR) was used, which contains the dual-frequency ZED-F9P GNSS chip from u-blox [8,9]. The ZED-F9P can receive satellite signals in both frequencies (F1 (1575.42 MHz) and F2 (1227.60 MHz)) from all available satellite constellations [8]. In F1 frequency those devices can track L1 C/A, L1OF, E1, and B1l signals while in F2 frequency, L2C, L2OF, E5b, and B2l signals. The simple RTK2B V1 board was used in combination with the Survey calibrated low-cost antenna offered by Ardusimple (denoted as LC−1 below), which has a phase center error of 1 mm and known calibration parameters from NGS (Figure 1) [9]. Another simpleRTK2B V1 board was used in combination with a geodetic antenna (JAV_RINGANT_G3T) from the Javad manufacturer (San Jose, CA, USA) denoted as LC−2 below). We used a high-quality GNSS geodetic instrument as a reference, Leica GS18T receiver and LEISGS 18 antenna, denoted as GD−1 below (Wetzlar, Germany). The geodetic GNSS instrument was used for the comparison of GNSS data, as well as to determine the coordinates of all points (described below) that were used as reference values.

### 2.2. Study Area

**Figure 2:**
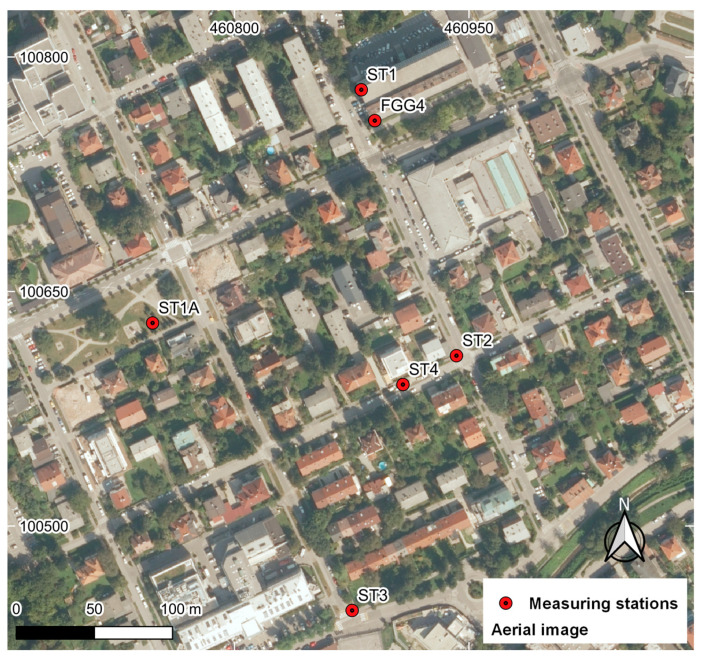
Measuring stations.

**Figure 3:**
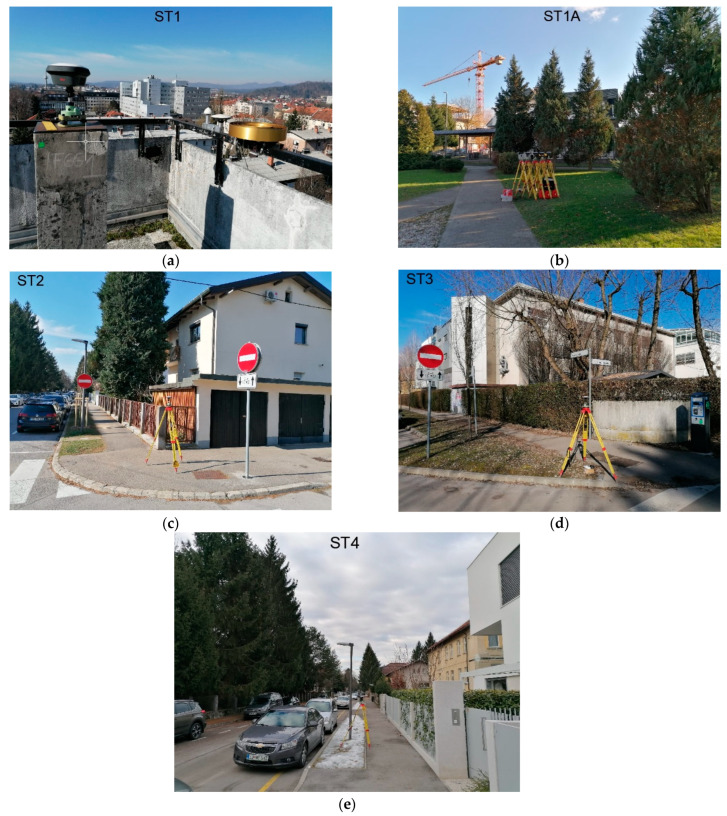
Location of measuring stations: (**a**) LC−1, LC−2, and GD−1 in ST 1; (**b**) LC−1, LC−2, and GD−1 in ST 1A; (**c**) LC−1 in ST 2; (**d**) LC−1 in ST 3; and (**e**) LC−1 in ST 4.

The study area was selected near the building of the Faculty of Civil and Geodetic Engineering of the University of Ljubljana (UL FGG), which offers both open-sky and urban conditions for conducting the observations. Five measuring stations were selected with positions shown in Figure 2. Station 1 (ST 1) is located on the rooftop of UL FGG and has open-sky conditions, while station 1A (ST 1A) is surrounded by trees (10 m) and buildings (Figure 3). GNSS observations from these two stations are used to verify the observation quality in open-sky and urban areas.

Station 2 (ST 2) and station 4 (ST 4) were 1–5 m from nearby buildings, which is often a situation in land-surveying tasks. Station 3 (ST 3) was more open to the horizon from the south side than the previous two stations (Figure 3). The observations of these three stations are used to evaluate the positioning performance of low-cost GNSS equipment in urban areas.

### 2.3. Quality Control of GNSS Observations

Low-cost GNSS devices are of lower quality than high-class geodetic GNSS instruments, the latter being considerably more expensive. Low-cost GNSS instruments have poorer design and, consequently, a lower carrier-to-noise ratio (C/N_0_). To better understand and explain the quality of observations from low-cost GNSS receivers, simultaneous GNSS observations with both types of devices in open-sky (ST 1) and urban areas (ST 1A) were obtained. One simpleRTK2B V1 receiver was connected to a low-cost GNSS antenna (LC−1), and the other simpleRTK2B V1 receiver to a geodetic GNSS antenna (LC−2). GNSS data from geodetic GNSS devices (GD−1) were used as a reference. Observations were collected for 24 h under open-sky conditions, followed by 4 h under urban conditions.

For a GNSS geodetic receiver, the C/N_0_ can vary from 35–45 dBHz, and this value can be less than 30 dBHz for signals received at low elevation angles, while a high-quality antenna can reach up to 50 dBHz. The values of C/N_0_ were determined for different elevation angles for LC−1, LC−2, and GD−1. Firstly, additional analyses were performed to show the variation of C/N_0_ as a function of the elevation angle for LC−1. Secondly, the average value of C/N_0_ was estimated on a basis of an additional 24 h set of GNSS observations obtained with a sampling rate of 1 Hz.

Low-cost GNSS antennas are more sensitive to multipath error compared to geodetic antennas, which can affect the quality of position determination, especially in urban areas [2]. Considering that the observations were performed at the same time and place, the differences in multipath presence between LC−1 and LC−2 are due to the different antennas used, while the differences with GD−1 are due to both the receiver and the antenna. The RMSE value of the code multipath (CMC) for which the differences are more visible was obtained for both cases and all types of GNSS instruments used.

The ambiguity fix ratio is another factor analyzed and compared between LC−1 and LC−2. In urban conditions, the multipath and other factors (cycle slips, loss of lock) may degrade the percentage of fix solutions, which results in positioning with low quality. For the open-sky and urban conditions, the percentage of fix ambiguities is shown for 24 h and 4 h, respectively. In addition, the determined coordinates and their variation for horizontal and vertical components are also presented graphically.

### 2.4. Positioning Performance of Low-Cost GNSS Devices in Urban Areas

To evaluate the positioning performance quality of low-cost GNSS devices in urban areas, observations performed in ST 1, ST 2, ST 3, and ST 4 were used. The data were acquired in each station for 1 h, which presents one session. This interval was divided into independent sub-session of 30 min, 20 min, and 15 min. Short sessions are considered to analyze the accuracy that can be achieved if a certain position is measured for a short time interval, which is the case in most surveying projects. For each session, horizontal and vertical coordinates $\left(e_{i},n_{i},h_{i}\right)$ were estimated in the Slovenian national coordinate system [29], while their differences from the true coordinates ($e_{t},n_{t},h_{t})$ were calculated as follows:

(1) $$ƛe=e_{i}-e_{t}$$

(2) $$ƛn=n_{i}-n_{t}$$

(3) $$ƛh=h_{i}-h_{t}$$

Reference coordinates were obtained from observations with geodetic GNSS devices (GD−1). The absolute differences for horizontal and spatial positions are estimated as follows:

(4) $$d_{2D}=\sqrt{{\left(e_{i}-e_{t}\right)}^{2}+{\left(n_{i}-n_{t}\right)}^{2}}$$

(5) $$d_{3D}=\sqrt{{\left(e_{i}-e_{t}\right)}^{2}+{\left(n_{i}-n_{t}\right)}^{2}+{\left(h_{i}-h_{t}\right)}^{2}}$$

To emphasize the differences between open-sky and urban locations, the ratio of ambiguities (fix or float) is shown. As a reference station, another low-cost GNSS device (as LC−1) was used, which was placed on point FGG4 with favorable conditions for the GNSS survey. The data processing was performed with open-source software RTKLIB (demo5_33b), and the processing settings are shown in Table 1 [30].

The RTK method is usually used for land surveying because it is not time-consuming and does not require post-processing. In this test, LC−1 was used as the rover, while the GSR1 station located 4 km away, which is part of the Slovenian CORS, served as the reference station for the corrections. Observations lasted 8 h at each station, and a total of 24 sessions were conducted. In each session, a triplet of coordinates was obtained, with a time difference of 20 min between sessions to account for changes in satellite geometry. First, the RMSE of the determined coordinates was estimated to determine the precision of the estimated coordinates. To analyze the positioning accuracy of low-cost receivers, the absolute differences for the horizontal and spatial positions were estimated similarly to the relative positioning explained above.

## 3. Results and Discussion

This section presents the results from the previous section. The quality test results of observations from low-cost GNSS devices are shown in Section 3.1, while the results of positioning performance of the same equipment in urban areas are presented in Section 3.2.

### 3.1. Results from Quality Control of GNSS Observations

#### 3.1.1. Carrier-to-Noise (C/N_0_) Analysis Results

**Figure 4:**
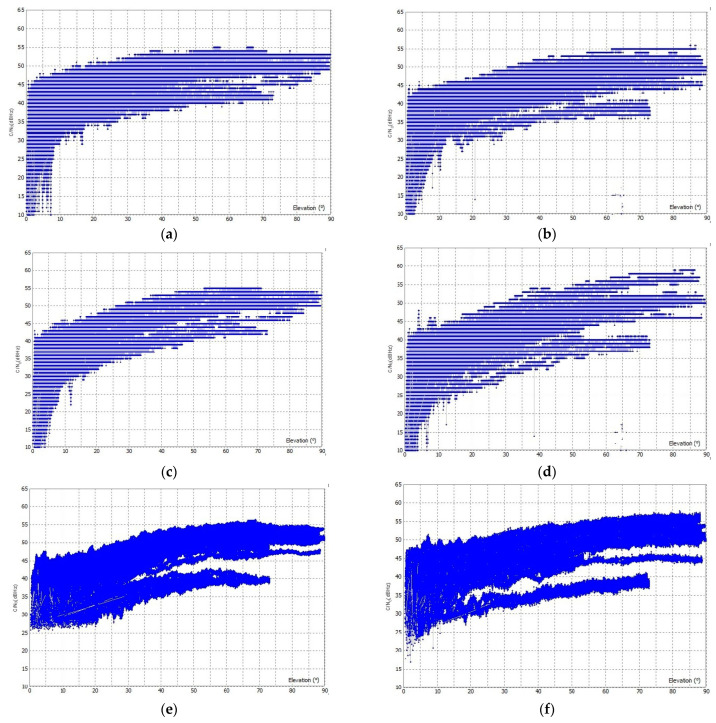
C/N_0_ for observations in F1 and F2 frequency in the open sky: (**a**) F1 C/N_0_ for LC−1, (**b**) F2 C/N_0_ for LC−1, (**c**) F1 C/N_0_ for LC−2, (**d**) F2 C/N_0_ for LC−2, (**e**) F1 C/N_0_ for GD−1, and (**f**) F2 C/N_0_ for GD−1.

All three GNSS instruments were placed side by side with only 20 cm between the antennas to assure the same surveying conditions. In the case of the open-sky conditions, GD−1 has a higher C/N_0_ for the observations in F1 frequency than LC−1 and LC−2, which is in the range of 25 to 55 dBHz (Figure 4), even at elevation angles lower than 10°. For LC−1 and LC−2, the C/N_0_ is greater than 40 dBHz at high elevation angles, while it is less than 25 dBHz at low elevation angles. The C/N_0_ for F2 is similar to F1; again GD−1 has higher C/N_0_ values than LC−2 and LC−1. The C/N_0_ for LC−1 and LC−2 is lower than 25 dBHz at elevation angles lower than 15° in both F1 and F2, while it is higher in the case of GD−1.

**Figure 5:**
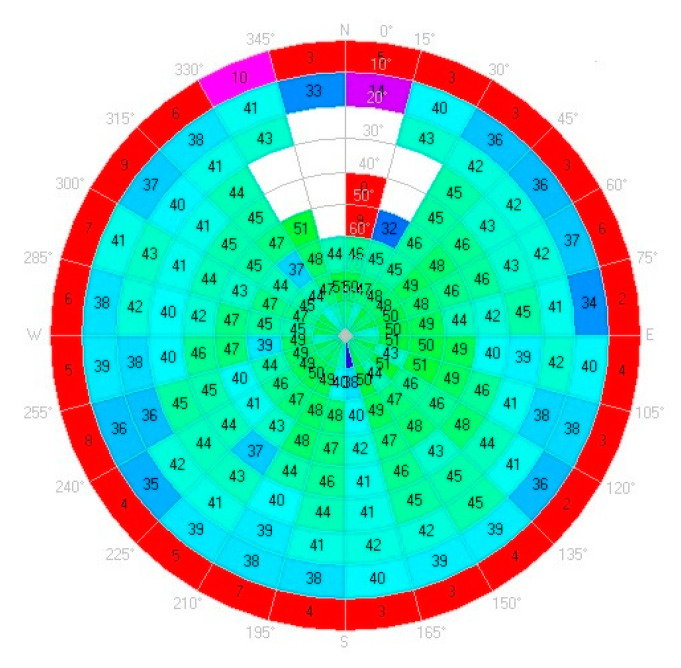
Sky plot of C/N_0_ for LC−1.

The LC−1 was additionally analyzed for its C/N_0_ in the open sky for 24 h using the open-source application u-center [31]. From the results shown in Figure 5, the C/N_0_ at elevation angles below 10° is less than 10 dBHz, while the C/N_0_ at elevation angles above 10° is more than 30 dBHz. The overall average C/N_0_ is 43.5 dB Hz with a deviation unit of 0.9 dBHz.

**Figure 6:**
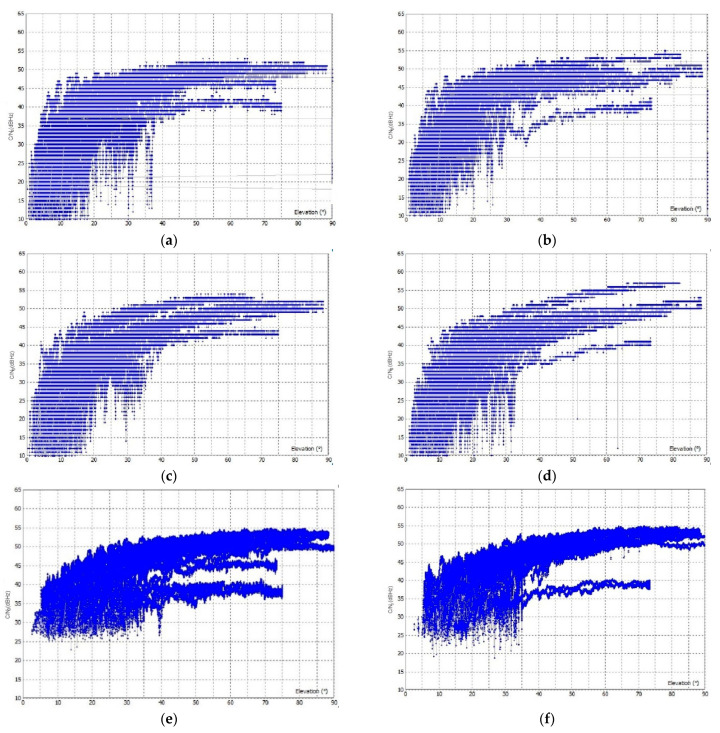
C/N_0_ for observations in F1 and F2 frequency in the urban areas: (**a**) F1 C/N_0_ for LC−1, (**b**) F2 C/N_0_ for LC−1, (**c**) F1 C/N_0_ for LC−2, (**d**) F2 C/N_0_ for LC−2, (**e**) F1 C/N_0_ for GD−1, and (**f**) F2 C/N_0_ for GD−1.

In urban areas, worse results are achieved with lower C/N_0_; this is especially true for low-cost GNSS devices (Figure 6). A C/N_0_ of 25 dBHz is obtained at elevation angles of 30° for LC−1 and LC−2 in both F1 and F2, while the C/N_0_ in open sky ranges between 30–35 dBHz at angles of 30°. The use of a geodetic antenna does not significantly improve the C/N_0_ for low-cost GNSS devices in urban areas. High-quality geodetic GNSS devices are found to perform better than low-cost ones; C/N_0_ is higher than 25 dBHz at lower elevation angles (<20°) for GD−1, while for LC−1 and LC−2, C/N_0_ is always less than 25 dBHz for both F1 and F2.

#### 3.1.2. Multipath Analysis

Multipath error is another important indicator that can influence the positioning solution, especially in urban areas. Low-cost antennas are more sensitive to multipath effects due to their quality and design, so the use of a ground plane is usually recommended. To better analyze the difference and the impact of the antenna, we used the same receiver in combination with the geodetic and the low-cost antenna, while a geodetic GNSS receiver was used as a reference for comparison. 

**Figure 7:**
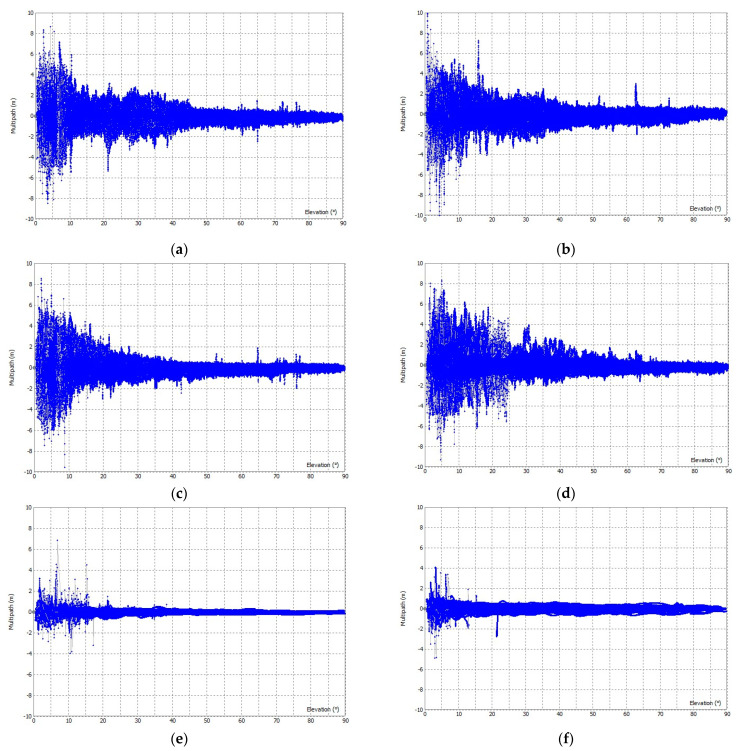
Multipath for code observations in F1 and F2 frequency in the open sky: (**a**) F1 multipath for LC−1, (**b**) F2 multipath for LC−1, (**c**) F1 multipath for LC−2, (**d**) F2 multipath for LC−2, (**e**) F1 multipath for GD−1, and (**f**) F2 multipath for GD−1.

**Figure 8:**
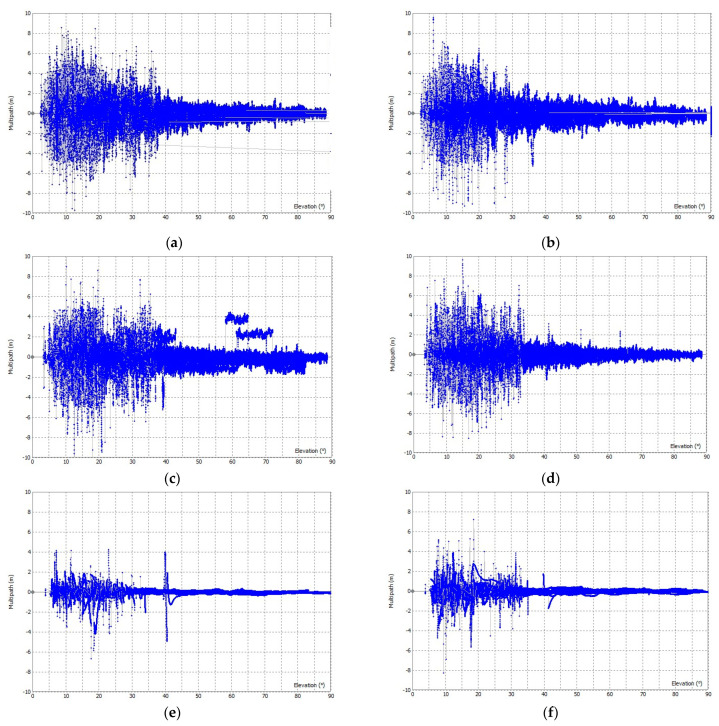
Multipath for code observations in F1 and F2 frequency in urban areas: (**a**) F1 multipath for LC−1, (**b**) F2 multipath for LC−1, (**c**) F1 multipath for LC−2, (**d**) F2 multipath for LC−2, (**e**) F1 multipath for GD−1, and (**f**) F2 multipath for GD−1.

The code multipath error for the open sky is shown graphically in Figure 7 and for urban conditions in Figure 8. The RMSE values of the multipath for both scenarios are shown in Table 2. Results show that LC−1 and LC−2 have very similar performances both in open-sky and in urban conditions. The use of a geodetic antenna does not significantly improve the performance of low-cost GNSS receivers in any scenario. The RMSE for LC−1 and LC−2 is worse in urban conditions than in open-sky conditions, which has more influence than the type of antennas used.

For the geodetic GNSS devices used (GD−1), the RMSE in the open sky is two times better than for LC−1 and LC−2, while in urban areas with more multipath error, the RMSE for GD−1 is 2–4 times better. The difference in RMSE for GD−1 under both conditions is not as significant as for LC−1 and LC−2.

#### 3.1.3. Ambiguity Resolution Results

The ambiguity fix ratio is a key factor for a high-precision positioning solution when phase observations are considered. The multipath error can affect the ambiguity fix ratio and provide float instead of fix solutions. This can be more evident when using low-cost GNSS equipment in urban areas.

**Figure 9:**
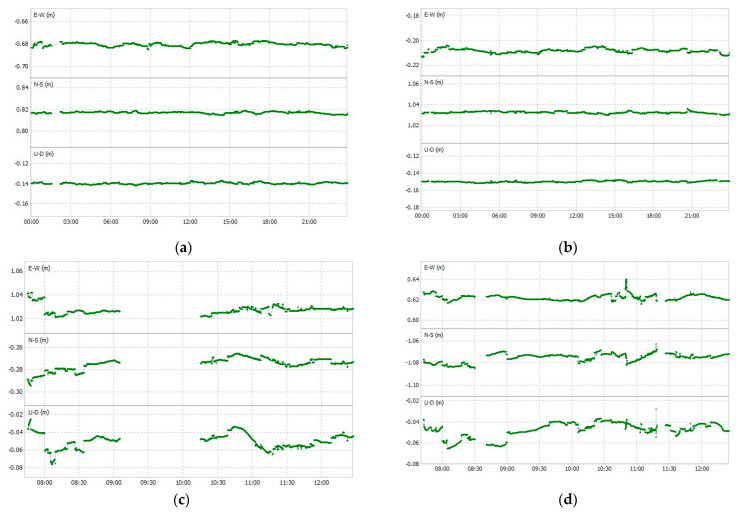
Positioning solutions in open sky and urban conditions: (**a**) LC−1 on ST 1 (open sky); (**b**) LC−2 on ST 1 (open sky); (**c**) LC−1 on ST 1A (urban areas); and (**d**) LC−2 on ST 1A (urban areas).

The ambiguity resolution ratio results are shown in Table 3 for LC−1 and LC−2. In the open sky, with less multipath and higher values of C/N_0_, the ambiguity fix ratio is more than 95%, LC−2 achieves an ambiguity fix ratio of 98.3%, while for LC−1 it is 96.8%. The difference is more obvious in urban areas, where the LC−2 has an ambiguity fix ratio of 93.4%, while it is only 75.0% for LC−1. Using a geodetic antenna improves the ambiguity fix ratio and provides fix solutions most of the time. In the case of the low-cost antenna, for intervals longer than 1 h, only float solutions are obtained. This may indicate the inappropriateness of using low-cost devices in similar conditions. The obtained positioning solutions for easting, northing, and ellipsoid height are shown for LC−1 and LC−2 in Figure 9 for both open-sky as well as urban conditions. In the open sky, fix solutions are obtained most of the time, while in urban areas more float solutions are present, which are shown with gaps in Figure 9.

### 3.2. Relative Positioning Performance 

The positioning performance of low-cost GNSS receivers in combination with Survey calibrated antenna (LC−1) was tested in urban areas, considering sessions of 60, 30, 20, and 15 min. Three of the stations were located in areas with obstacles in the vicinity (ST 2, ST 3, and ST 4), while one of them (ST 1) was in the open sky to highlight the differences. For each of the stations, the differences in the obtained coordinates (easting, northing, and ellipsoid height) from the true coordinates are estimated for sessions of 15–60 min. The horizontal (d_2D_) and spatial differences (d_3D_) between the estimated and true coordinates are also presented for each station.

The results for ST 1 are shown in Table 4. Based on the obtained results, it can be concluded that low-cost GNSS receivers perform well in the open sky. The horizontal, vertical, and spatial position differences are less than 5 mm, and these results are valid even for short sessions lasting 15 min. The ambiguity fix ratio for the main session is 99.5%, and LC−1 can provide fix solutions in all the sessions considered.

ST 2 was located in an urban area with obstacles/buildings nearby where higher influence of multipath was expected than in ST 1. For the horizontal positions, the differences are less than 10 mm in 80% of the sessions, while the differences in spatial positions are between 5–30 mm (Table 5). For the 60 min session, the ambiguity rate of the fixed solutions is 64.8% under these conditions, even though a short baseline is considered. Compared to ST 1, the positioning performance in ST 2 is worse, especially for the spatial component. This was due to the accuracy obtained for the ellipsoid height, which was determined with lower accuracy compared to the horizontal components.

ST 3 has fewer obstacles from the south side than ST 2 and obtained results are shown in Table 6. The horizontal position differences do not exceed 10 mm even in the short sessions, while the differences in the spatial and vertical positions remain below 15 mm. In comparison to the previous station, better accuracy is achieved for the vertical component, while the ambiguity fix ratio is 90.3%, which is 25.5% higher than for ST 2 (64.8%).

ST 4 is surrounded by tall trees (10 m) on the south side, therefore, poorer results are obtained, which are listed in Table 7. The sub-sessions of 40–60 min, as well as sub-sessions 45–60 min, do not provide fix solutions. In these two sessions, the accuracy exceeds 10 cm, therefore, these values are not shown. Nevertheless, for sessions with fix solutions, the horizontal and spatial differences are below 15 and 25 mm, respectively, while the vertical position differences are less than 20 mm. For this station with more obstacles for satellite signals, the percentage of ambiguity fix ratio is 51.6%, which is the lowest value for all stations.

**Figure 10:**
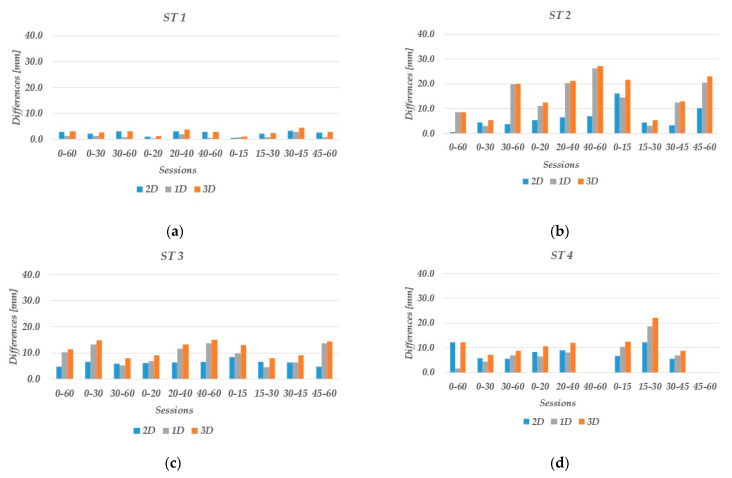
Horizontal, vertical, and spatial positioning accuracy in the open-sky (ST 1) and urban areas (ST 2, ST 3, ST 4): (**a**) LC−1 at ST 1; (**b**) LC−1 at ST 2; (**c**) LC−1 at ST 3; and (**d**) LC−1 at ST 4.

The results of the horizontal (d_2D_), vertical (ƛh), and spatial absolute (d_3D_) position differences shown in the last four tables are also presented graphically in Figure 10. In open-sky conditions, the differences are less than 5.0 mm for horizontal, vertical, and spatial positions in all sessions. Under urban conditions, buildings and trees near the stations degrade the signal quality, and low-cost instruments prove to have poorer positioning results. The differences for the horizontal and vertical positions are smaller than 10 and 15 mm in 85% and 82.5% of the sessions, respectively. For the spatial positions, the differences are smaller than 15 mm in 77.5% of the considered sessions in urban areas.

### 3.3. RTK Positioning Performance 

**Figure 11:**
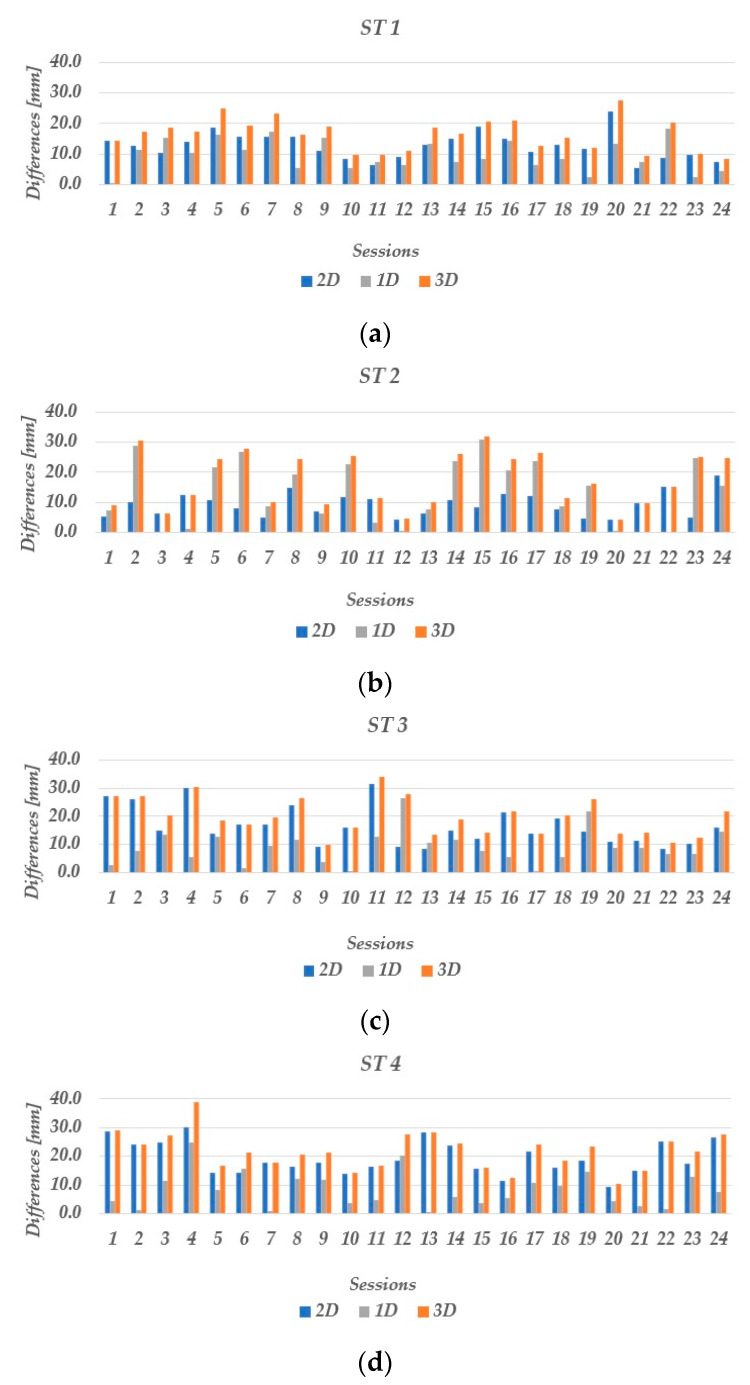
Horizontal, vertical, and spatial positioning accuracy in the open-sky (ST 1) and urban areas (ST 2, ST 3, ST 4) for 24 sessions: (**a**) LC−1 at ST 1; (**b**) LC−1 at ST 2; (**c**) LC−1 at ST 3; and (**d**) LC−1 at ST 4.

Low-cost dual-frequency GNSS receivers were tested in RTK in both open-sky and urban areas. Firstly, the RMSE for easting, northing, and ellipsoid height was estimated, then the horizontal and spatial RMSE were obtained for all stations by considering 24 sessions (Table 8). From the obtained results, it can be concluded that the horizontal and spatial RMSE in the open sky (ST 1) is less than 1 cm. Similar results were obtained for other stations (ST 2, ST 3, and ST 4) in urban areas, where the horizontal and spatial RMSE does not exceed 2 cm. To evaluate the quality of positioning of low-cost GNSS receivers in RTK mode, the positioning accuracy was estimated as the absolute difference in horizontal (d_2D_), vertical (ƛh), and spatial absolute (d_3D_) positions from the true position (Figure 11).

At ST 1, in the open sky, a horizontal positioning accuracy of 2 cm is achieved in 96% of the sessions and a spatial positioning accuracy of 2 cm in 75% of the sessions. At other stations (ST 2, ST 3, and ST 4) located in urban areas with obstacles to satellite signals, the horizontal positioning accuracy of 2 cm is achieved in 100%, 75%, and 62.5% of the sessions for ST 2, ST 3, and ST 4, respectively. The spatial accuracy is less than 2 cm in 54.2% of the sessions for ST 2 and ST 3, while for ST 4 this stands true only in 37.5% of the sessions. In addition to the statistics presented, it can be highlighted that the spatial accuracy of low-cost GNSS receivers under open-sky conditions is less than 3 cm in all sessions, while in the case of urban conditions, an accuracy of 3 cm is achieved in 95% of the sessions.

## 4. Conclusions

Low-cost GNSS receivers with calibrated low-cost and geodetic antennas were compared to geodetic GNSS devices in urban areas where various obstacles can affect the quality of the received signals. Therefore, the C/N_0_, multipath effect, and ambiguity fix ratio were analyzed under both conditions, open-sky and urban, highlighting the differences between the devices/antennas used. The positioning performance of low-cost devices was also tested in urban areas at different locations with and without buildings/obstacles nearby. The results from this study lead us to the following conclusions:

- Low-cost GNSS devices have lower C/N_0_ than high-quality geodetic GNSS devices in both open-sky and urban areas. In the first case, C/N_0_ is below 25 dBHz at elevation angles lower than 15°, whereas the C/N_0_ remains above 25 dBHz for low elevations in the case of geodetic devices. In urban areas, the C/N_0_ is greater than 25 dBHz at elevation angles below 20° for geodetic instruments, whereas C/N_0_ is always below 25 dBHz for low-cost GNSS equipment. The urban conditions affect low-cost GNSS devices more than geodetic devices;
- Low-cost GNSS instruments exhibit a multipath RMSE two times higher than geodetic devices in the open sky, while this difference is up to four times higher in urban areas. Geodetic GNSS devices show almost the same results under both conditions, where the multipath worsens significantly for low-cost devices and urban areas, even when a geodetic antenna is used;
- The ambiguity fix ratio is more than 95% for low-cost devices in the open sky in combination with a low-cost and geodetic antenna;
- In urban areas, the low-cost GNSS antenna performs worse than the geodetic antenna, and the ambiguity fix ratio is 18.4% lower for the low-cost GNSS antenna used;
- In the open sky, low-cost GNSS receivers with calibrated low-cost antennas achieve a horizontal and spatial positioning accuracy of 5 mm over short baselines even if short sessions (15 min) are considered;
- In urban areas with surrounding obstacles, horizontal accuracy is better than 10 mm in 85% of sessions, while spatial accuracy is better than 15 mm in 77.5% of sessions. For the vertical component, the accuracy is smaller than 15 mm in 82.5 % of sessions;
- Low-cost GNSS sensors are more sensitive to multipath and other error sources that degrade the quality of position determination in urban areas. Consequently, fix solutions were not obtained in some sessions. However, considering their price and the demonstrated positioning performance, those GNSS sensors can be an alternative option to geodetic GNSS devices for surveying in static relative mode (e.g., establishing geodetic networks) in urban areas, especially in projects with a limited budget;
- Low-cost GNSS instruments can achieve 1–3 cm positioning accuracy in RTK mode in urban areas and are suitable to be used in many surveying projects.

In this study, all tests were performed over short baselines because the goal was to only evaluate the performance of double-frequency low-cost GNSS receivers in urban areas, as these devices are more sensitive to different error sources that can degrade the positioning quality. The results presented in this paper should be considered as preliminary. A more detailed analysis will be performed in the future when we will test these devices in RTK mode over longer baselines.

## Figures and Tables

**Table 1:** Used parameters in the data processing.

Parameters	RTKLIB
Observations	Phase
Duration	1 h, 30 min, 20 min, and 15 min
Constellations	GPS, GLONASS, Galileo
Ambiguity	Continuous
Elevation mask	15°

**Table 2:** RMSE for multipath error in open-sky and urban conditions.

Scenarios	Frequency	LC−1	LC−2	GD−1
Open sky	F1	0.50 m	0.51 m	0.16 m
	F2	0.48 m	0.57 m	0.23 m
Urban area	F1	0.86 m	0.90 m	0.23 m
	F2	0.76 m	0.70 m	0.31 m

**Table 3:** Ambiguity resolution ratio for LC−1 and LC−2.

Scenarios	Solution	LC−1	LC−2
Open sky	Fixed	96.8%	98.3%
	Float	3.2 %	1.7%
Urban area	Fixed	75.0%	93.4%
	Float	25.0%	6.6%

**Table 4:** Positioning performance results at ST 1.

Sessions (min)	ƛe (mm)	ƛn (mm)	ƛh (mm)	d_2D_ (mm)	d_3D_ (mm)
0–60	0.7	−2.8	−1.3	2.9	3.2
0−30	0.7	−2.1	−1.2	2.2	2.5
30–60	0.7	−2.9	−0.9	3.0	3.1
0−20	0.1	−1.1	−0.4	1.1	1.2
20–40	1.0	−3.0	−1.9	3.2	3.7
40−60	0.6	−2.7	−0.6	2.8	2.8
0–15	0.2	−0.5	−0.9	0.5	1.0
15−30	0.6	−2.2	−0.7	2.3	2.4
30–45	1.8	−2.9	−2.9	3.4	4.5
45−60	0.6	−2.6	−0.7	2.7	2.8

**Table 5:** Positioning performance results at ST 2.

	ƛe (mm)	ƛn (mm)	ƛh (mm)	d_2D_ (mm)	d_3D_ (mm)
0–60	−0.5	0.0	8.5	0.5	8.5
0–30	−4.3	−0.9	3.0	4.4	5.3
30–60	3.2	−2.1	19.8	3.8	20.2
0–20	−5.0	2.1	11.2	5.4	12.4
20–40	−2.4	−6.0	20.3	6.5	21.3
40–60	5.4	−4.5	26.3	7.0	27.2
0–15	−5.5	−15.3	14.5	16.3	21.8
15–30	−4.3	−0.9	3.0	4.4	5.3
30–45	−3.3	0.7	12.5	3.4	12.9
45–60	10.1	−0.8	20.6	10.1	23.0

**Table 6:** Positioning performance results at ST 3.

Sessions (min)	ƛe (mm)	ƛn (mm)	ƛh (mm)	d_2D_ (mm)	d_3D_ (mm)
0–60	−4.8	0.4	10.3	4.8	11.4
0−30	−6.6	−0.9	13.3	6.7	14.9
30–60	−4.8	3.5	5.2	5.9	7.9
0–20	−6.0	0.6	6.8	6.0	9.1
20–40	−3.8	5.2	−11.6	6.4	13.3
40–60	−6.4	−1.3	13.6	6.5	15.1
0–15	−6.9	−4.9	−9.8	8.5	12.9
15–30	−5.3	3.9	4.6	6.6	8.0
30–45	−6.3	0.7	6.4	6.3	9.0
45–60	−4.6	1.2	13.6	4.8	14.4

**Table 7:** Positioning performance results at ST 4.

Sessions (min)	ƛe (mm)	ƛn (mm)	ƛh (mm)	d_2D_ (mm)	d_3D_ (mm)
0–60	−8.8	8.4	−1.5	12.2	12.3
0–30	−5.2	2.4	4.3	5.7	7.2
30–60	−3.3	4.4	6.9	5.5	8.8
0–20	−8.3	0.3	−6.5	8.3	10.5
20–40	−7.4	5.0	−8.1	8.9	12.1
40–60	/	/	/	/	/
0–15	−6.1	2.7	10.4	6.7	12.4
15–30	−10.7	5.7	−18.6	12.1	22.2
30–45	−3.3	4.4	6.9	5.5	8.8
45–60	/	/	/	/	/

**Table 8:** RTK positioning precision in the open-sky (ST 1) and urban areas (ST 2, ST 3, and ST 4).

Station	e (mm)	n (mm)	h (mm)	d_2D_ (mm)	d_3D_ (mm)
ST1	3.4	5.3	5.1	6.4	8.1
ST2	6.5	6.5	13.5	9.2	16.3
ST3	7.2	6.7	7.3	9.8	12.2
ST4	6.0	8.4	10.0	10.3	14.4

## Data Availability

The data presented in this study are available on request from the corresponding author.
